# Supplementary material for: Resolving the full spectrum of human genome variation using Linked-Reads
Source: Genome Res. 2019 Apr;29(4):635–45. doi: 10.1101/gr.234443.118 (PMC6442396; doi:10.1101/gr.234443.118)
Supplement: Supplemental Material [file supp_gr.234443.118_Supplemental_longranger-2.2.zip › longranger-2.2.tar.bz2!/lr2.2-public/lib/stan/cmdstan/stan/doc/api/html/arg__test__gradient_8hpp_source.html]

Stan: src/stan/gm/arguments/arg\_test\_gradient.hpp Source File


|  |  |
| --- | --- |
|  | Stan  2.5.0  probability, sampling & optimization |


- Main Page
- Related Pages
- Namespaces
- Classes
- Files

- File List
- File Members

All Classes Namespaces Files Functions Variables Typedefs Enumerations Enumerator Friends Macros Pages

- src
- stan
- gm
- arguments

arg\_test\_gradient.hpp

Go to the documentation of this file.

1 #ifndef STAN\_\_GM\_\_ARGUMENTS\_\_TEST\_\_GRADIENT\_\_HPP

2 #define STAN\_\_GM\_\_ARGUMENTS\_\_TEST\_\_GRADIENT\_\_HPP

3

4 #include <stan/gm/arguments/categorical\_argument.hpp>

5 #include <stan/gm/arguments/arg\_test\_grad\_eps.hpp>

6 #include <stan/gm/arguments/arg\_test\_grad\_err.hpp>

7

8 namespace stan {

9

10  namespace gm {

11

12  class arg\_test\_gradient: public categorical\_argument {

13

14  public:

15

16  arg\_test\_gradient() {

17

18  \_name = "gradient";

19  \_description = "Check model gradient against finite differences";

20

21  \_subarguments.push\_back(new arg\_test\_grad\_eps());

22  \_subarguments.push\_back(new arg\_test\_grad\_err());

23

24  }

25

26  };

27

28  } // gm

29

30 } // stan

31

32 #endif

33

stan::gm::arg\_test\_gradient

**Definition:** arg\_test\_gradient.hpp:12

arg\_test\_grad\_err.hpp

stan::gm::arg\_test\_gradient::arg\_test\_gradient

arg\_test\_gradient()

**Definition:** arg\_test\_gradient.hpp:16

categorical\_argument.hpp

stan::gm::categorical\_argument

**Definition:** categorical\_argument.hpp:11

stan::gm::categorical\_argument::\_subarguments

std::vector< argument \* > \_subarguments

**Definition:** categorical\_argument.hpp:153

stan::gm::argument::\_name

std::string \_name

**Definition:** argument.hpp:76

stan::gm::arg\_test\_grad\_err

**Definition:** arg\_test\_grad\_err.hpp:10

arg\_test\_grad\_eps.hpp

stan::gm::arg\_test\_grad\_eps

**Definition:** arg\_test\_grad\_eps.hpp:10

stan::gm::argument::\_description

std::string \_description

**Definition:** argument.hpp:77


---

[ Stan Home Page ]

*© 2011–2014,
Stan Development Team.*
